# Defence Signalling Triggered by Flg22 and Harpin Is Integrated into a Different Stilbene Output in *Vitis* Cells

**DOI:** 10.1371/journal.pone.0040446

**Published:** 2012-07-06

**Authors:** Xiaoli Chang, Peter Nick

**Affiliations:** Molecular Cell Biology, Botanical Institute 1, Karlsruhe Institute of Technology, Karlsruhe, Germany; University of Wisconsin-Milwaukee, United States of America

## Abstract

Plants can activate defence to pathogen attack by two layers of innate immunity: basal immunity triggered by pathogen-associated molecular pattern (PAMP) triggered immunity (PTI) and effector-triggered immunity (ETI) linked with programmed cell death. Flg22 and Harpin are evolutionary distinct bacterial PAMPs. We have previously shown that Harpin triggers hypersensitive cell death mimicking ETI in *Vitis rupestris*, but not in the *Vitis vinifera* cultivar ‘Pinot Noir’. In contrast, the bacterial PAMP flg22 activating PTI does not trigger cell death. To get insight into the defence signalling triggered by flg22 and Harpin, we compared cellular responses upon flg22 and Harpin treatment in the two *Vitis* cell lines. We found that extracellular alkalinisation was blocked by inhibition of calcium influx, and modulated by pharmacological manipulation of the cytoskeleton and mitogen-activated protein kinase activity with quantitative differences between cell lines and type of PAMPs. In addition, an oxidative burst was detected that was much stronger and faster in response to Harpin as compared to flg22. In *V. rupestris*, both flg22 and Harpin induced transcripts of defence-related genes including *stilbene synthase*, microtubule disintegration and actin bundling in a similar way, whereas they differed in *V. vinifera* cv. ‘Pinot Noir’. In contrast to Harpin, flg22 failed to trigger significant levels of the stilbene *trans*-resveratrol, and did not induce hypersensitive cell death even in the highly responsive *V. rupestris*. We discuss these data in a model, where flg22- and Harpin-triggered defence shares a part of early signal components, but differs in perception, oxidative burst, and integration into a qualitatively different stilbene output, such that for flg22 a basal PTI is elicited in both cell lines, while Harpin induces cell death mimicking an ETI-like pattern of defence.

## Introduction

Plants employ two distinct layers of immunity to encounter pathogen invasion [1]. The first, evolutionarily ancient, layer involves the perception of conserved pathogen structures termed pathogen-associated molecular patterns (PAMPs) at the plasma membrane through conserved and ubiquitous receptors generally defined as pattern recognition receptors (PRRs). Binding to these receptors initiates an active defence response, so-called PAMP-triggered immunity (PTI), in both host and non-host plants. In a second round of host-pathogen warfare, several microbial pathogens have already developed the ability to secrete effector proteins into the cytoplasm using type-III secretion systems (T3SS) in bacteria. These effectors suppress PTI and result in the effector-triggered susceptibility (ETS) [2],[3]. In response to pathogen effectors, plants have acquired additional receptors that specifically recognise the effectors, establishing a second layer of immunity known as effector-triggered immunity (ETI). ETI is often associated with a hypersensitive response (HR), a plant-specific form of programmed cell death at the infection sites, in many cases followed by systemic acquired resistance (SAR) in the hosts. The dynamic and continuous co-evolution between the two opponents stimulates on side of the pathogen the formation of novel effectors to suppress the ETI response [4],[5]. On the side of the host, new plant resistance (R) proteins are developed to recognise the effectors to reconsolidate ETI [1],[6].

Typically, perception of PAMPs rapidly activates early defence responses including depolarisation of the plasma membrane [7], opening of ion channels [8],[9], activation of a mitogen-activated protein kinase (MAPK) cascades [10], generation of reactive oxygen species (ROS), reinforcement of the cell wall, transcription of defence genes, and phytoalexin accumulation [11],[12]. The best characterised PAMP is the peptide flg22, corresponding to the highly conserved N-terminal part of eubacterial flagellin, activating defence responses in most plant species [7]. Recognition of flg22 by the leucine-rich repeat (LRR) receptor kinase FLS2 [13],[14] leads to increased intracellular Ca^2+^ concentration, oxidative burst, activation of MAPKs, transcription of defence-related genes through the WRKY transcription factors WRKY22/29 and WRKY25/33, and ethylene biosynthesis [10],[15],[16].

While PAMPs are commonly considered to be essential for general microbial fitness and survival, effectors, secreted or injected into the plant cell [4], specifically contribute to pathogen virulence by affecting specific targets of the host, such as receptor kinases [17], ubiquitination [18], vesicle trafficking [19], cell-wall reinforcement [20], secretion of toxic plant proteins [21], and hypersensitive reaction (HR) [22]. Harpin proteins, first described in *Erwinia amylovora*, the causal agent for the fire-blight disease of apple, pear and other members of the Rosaceae [23], belong to a group of effector proteins exported by a bacterial T3SS and have been intensively studied for their ability to initiate hypersensitive cell death and to induce systemic acquired resistance [24]–[28]. When applied to non-host plants, Harpin triggers immunity-associated responses, such as ROS production [29],[30], accumulation of defence-related transcripts and cell death [31],[32],[33], Thus, Harpin proteins can mimick certain aspects of ETI.

The conceptual discrimination between PTI and ETI has been challenged by recent studies identifying transitions between PAMPs and effectors [34]. The activation of immune responses in PTI and ETI through PAMPs and effectors, and through different PAMPs appears to share common events [3]. Thus, the dichotomy might be not of qualitative, but of quantitative nature, and it might depend merely on magnitude and duration of the interactions among the components. Moreover, upon recognition of different pathogenic virulence factors, plants can trigger different responses, and, in addition, different disease-tolerant plants respond to specific pathogens differently [35],[36]. Therefore, the complex signalling resulting in PTI or ETI, and the similarities and differences among PTI responses triggered by different PAMPs warrants further investigation.

Grapevine, a widespread and important agricultural fruit crop, is affected by several diseases such as Downy and Powdery Mildew [37],[38]. During the long co-evolution with these pathogens, North American *Vitis* species have developed sophisticated and robust defence mechanism probably based on recognition of pathogen effectors by the products of host resistance (*R*) genes. In contrast, European grapevines have evolved without contact to these pathogens, and therefore represent naive hosts that lack effective mechanisms to limit pathogenic infection. As an important strategy to improve the resistance against pathogens without the need for expensive and ecologically problematic pesticides, the innate immunity of grapevine can be activated by pathogen-derived elicitors [39],[40],[41]. The success of this strategy depends on our understanding of the molecular and cellular signal mechanism underlying grapevine-pathogen interaction.

We therefore conducted a comparative analysis of early defence signalling triggered by two evolutionarily different bacterial PAMPs, flg22 versus Harpin, in suspension cells derived from the pathogen resistant North American species *Vitis rupestris* and the susceptible grapevine *Vitis vinifera* cultivar ‘Pinot Noir’. We investigated the dependence of apoplastic alkalinisation on calcium influx, MAPK cascades, and cytoskeleton, oxidative burst, expression of defence genes, biosynthesis of stilbenes, and cytoskeletal reorganisation, and arrive at a model, where early defence responses triggered by flg22 and Harpin partially overlap, but differ in perception and oxidative burst, which are integrated into a qualitatively different final output with respect to stilbene patterns and cell death. Whereas flg22 triggers a basal PTI in both cell lines, Harpin, although commonly accepted as a class of PAMPs due to its widespread distribution among the bacterial pathogens, triggers an ETI-like defence.

## Results

### Flg22-induced extracellular alkalinisation differs in two cell lines

One of the earliest responses detected is a modification of plasma membrane permeability, in particular, Ca^2+^, H^+^ and K^+^, and anion fluxes that can be conveniently followed as changes of extracellular pH [7],[42]. We therefore followed apoplastic alkalinisation after treatment with the bacterial PAMP flg22 to compare it with our previous data on the bacterial secreted protein Harpin [33].

**Figure 1:**
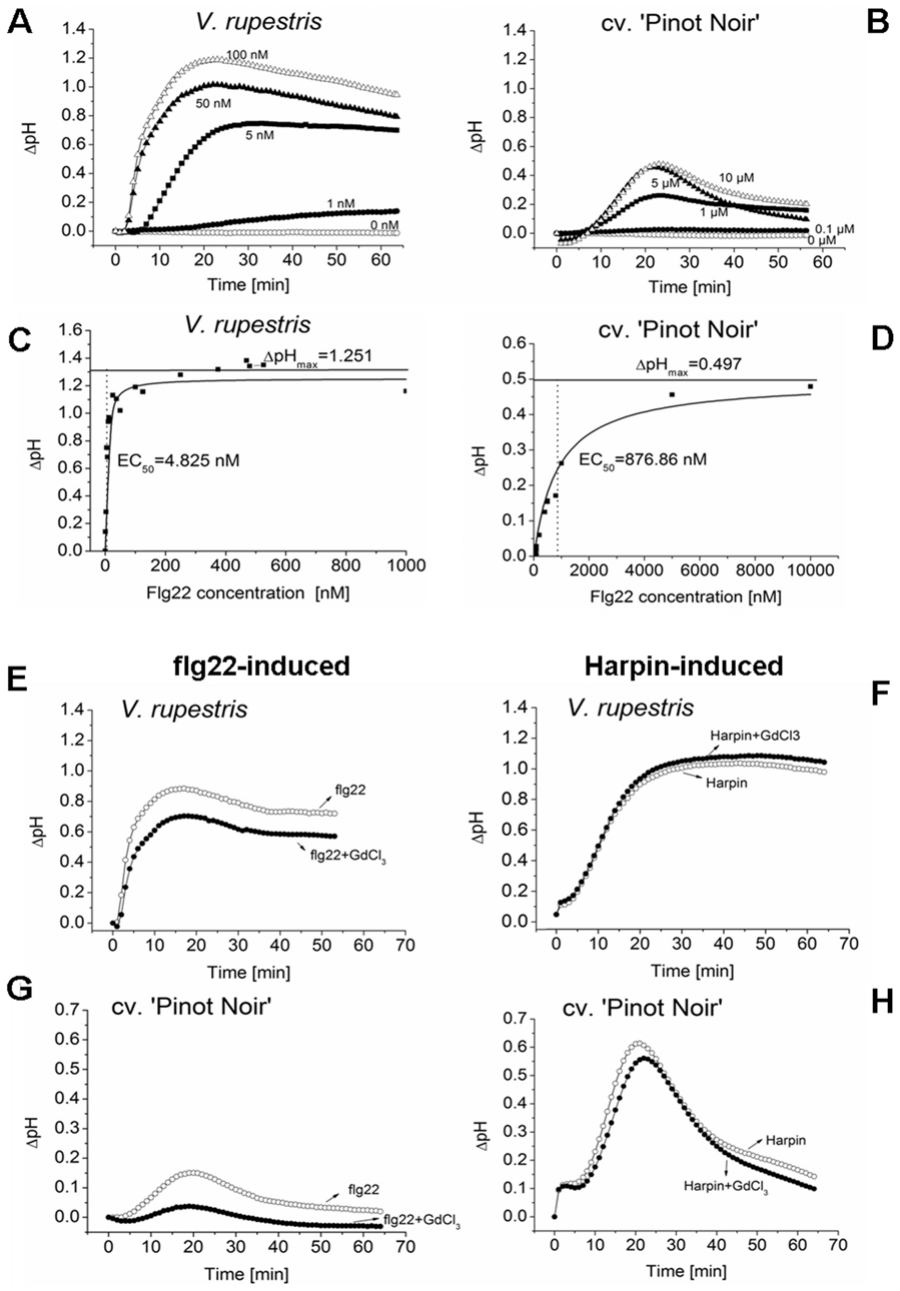
Apoplastic alkalinisation evoked by flg22 and Harpin in the two grapevine cell lines. **A, B** Dose response of extracellular alkalinisation to flg22 over time in *Vitis rupestris* (**A**) and *Vitis vinifera* cv. ‘Pinot Noir’ (**B**). **C, D** Analysis of the maximum change of extracellular pH in response to the increasing concentration of flg22. Data were fitted using a Michaelis-Menten equation [f (x) = ΔpH_max_×x/(EC_50_+x)], where ΔpH_max_ = 1.251 (*V. rupestris*) or 0.497 (*V. vinifera* cv. ‘Pinot Noir’), and EC_50_ = approximately 4.825 nM (*V. rupestris*) or 876.86 nM (*V. vinifera* cv. ‘Pinot Noir’), respectively. **E–H** Role of Gd-sensitive calcium channels for apoplastic alkalinisation induced by 1 µM flg22 (**E, G**) or 9 µg^.^ml^−1^ Harpin (**F, H**) in combination with the solvent DMSO (open circles) or with 20 µM of GdCl_3_ (closed circles) either in *V. rupestris* (E, F) or *V. vinifera* cv. ‘Pinot Noir’ (G, H), respectively. Representative timelines from five independent series are shown.

Extracellular pH increased rapidly from about 30 s after addition of flg22, culminated in about 20 min, and subsequently decreased slowly in *V. rupestris* (Figure 1A). In *V. vinifera* cv. ‘Pinot Noir’, the increase of pH initiated later (from 5 min), and the amplitude of the peak at 20 min was lower by a factor of 2 (Figure 1B). The magnitude of the peak was dependent on the concentration of flg22 (Figures 1A, B). We therefore compared the difference between the two cell lines on a quantitative level, and recorded numerous time-courses over different concentrations of flg22. The dependency of maximal ΔpH on the respective concentration of flg22 (Figures 1C, D) could be fitted using a Michaelis-Menten equation (R^2^ = 0.960 for *V. rupestris*; and R^2^ = 0.962 for *V. vinifera* cv. ‘Pinot Noir’), where effective concentrations (EC_50_, inducing 50% of the maximal response) could be calculated to be 4.825 nM in *V. rupestris* and 876.86 nM in *V. vinifera* cv. ‘Pinot Noir’ respectively. This means that the sensitivity of *V. rupestris* is roughly 200 times higher, compared with *V. vinifera* cv. ‘Pinot Noir’. Corresponding to EC_50_, ΔpH_max_ was approximately 1.251 in *V. rupestris* and 0.497 in *V. vinifera* cv. ‘Pinot Noir’. To establish a situation, where the pH response as readout for signal input was comparable between *V. rupestris* and *V. vinifera* cv. ‘Pinot Noir’, a concentration of 1 µM flg22 was used in the following experiments.

In our previous work, we had quantified the response to Harpin [33], and observed a similar difference in the sensitivity of the two cell lines. However, compared to elicitation with Harpin, the pH response triggered by flg22 was faster (maximum reached at about 20 min) than for Harpin (maximum reached at 30 min), indicating a more rapid signal transfer between binding of the elicitor and proton flux for flg22 as compared to Harpin.

### Flg22-induced extracellular alkalinisation is more sensitive to Gd ions

Extracellular alkalinisation records the activity of a calcium influx channel essential for the activation of early defence [43] and should be blocked by GdCl_3_, an inhibitor of mechanosensitive calcium channels [44]. We therefore measured extracellular alkalinisation evoked by flg22 and Harpin in presence of GdCl_3_ in *V. rupestris* (Figures 1E, F) and *V. vinifera* cv. ‘Pinot Noir’ (Figures 1G, H). In both cell lines, alkalinisation in response to flg22 was significantly inhibited by addition of 20 µM GdCl_3_ as compared to the solvent control (Figures 1E, G). In contrast to flg22, Harpin-triggered alkalinisation was not significantly affected by 20 µM GdCl_3_ (Figures 1F, H), indicating that Harpin-triggered pH change is not sensitive to Ca^2+^, and even a concentration as high as 1 mM GdCl_3_ inhibited Harpin-elicited alkalinisation only to a low extent. This finding suggests that Ca^2+^ influx through the plasma membrane is required for the alkalinisation induced by flg22, but is only indirectly linked to Harpin-triggered alkalinisation.

### Negative feedback of MAPK signalling on alkalinisation

**Figure 2:**
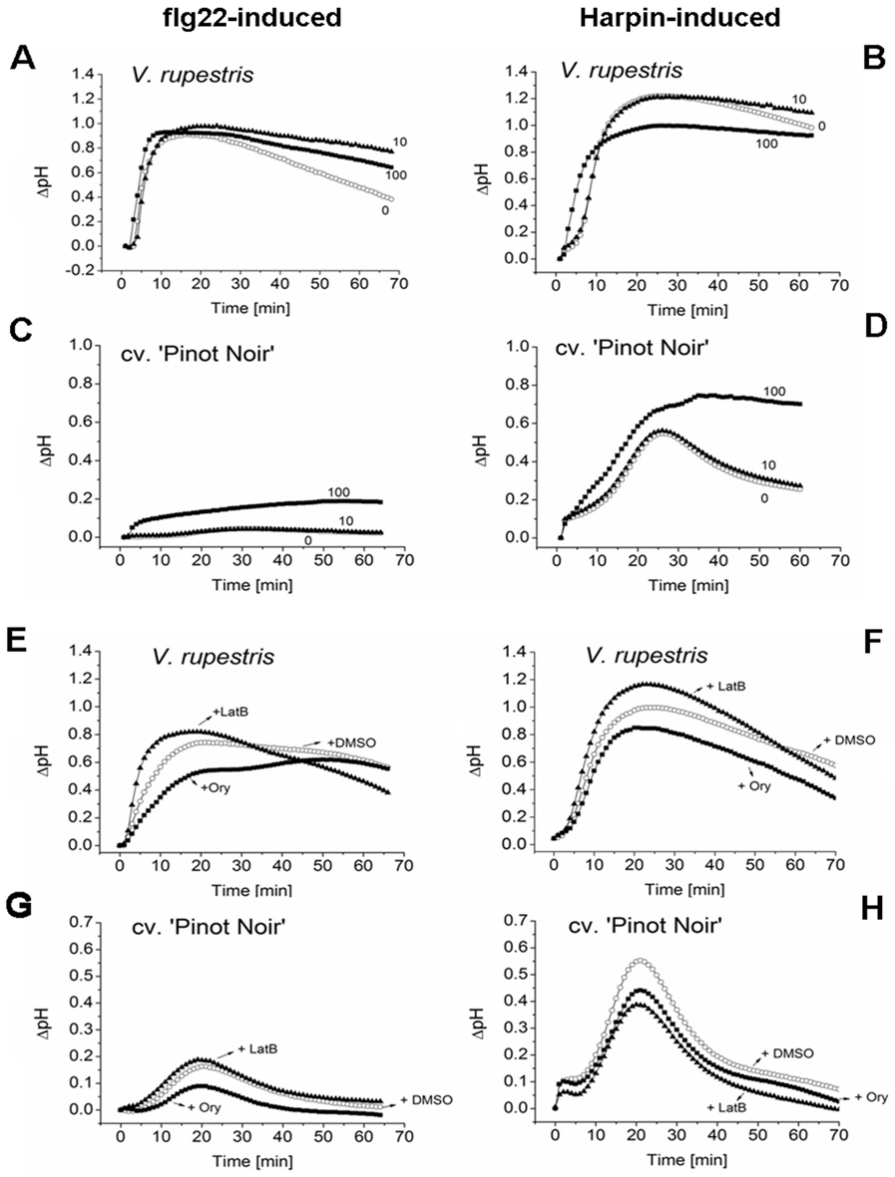
Feedback of downstream factors on flg22- and Harpin-induced apoplastic alkalinisation. **A–D** Effect of the MAPK cascades inhibitor PD98059 (PD) on flg22- and Harpin-dependent alkalinisation in *V. rupestris* (**A, B**) versus *V. vinifera* cv. ‘Pinot Noir’ (**C, D**). Cells were elicited by either 1 µM flg22 (**A, C**) or 9 µg^.^ml^−1^ Harpin (**B, D**) in combination with 0 µM (open circles), 10 µM (closed triangles), or 100 µM (closed squares) PD98059 (PD). **E–H** Effects of cytoskeletal drugs on flg22 and Harpin-dependent alkalinisation, respectively. Effects of the microtubule inhibitor Oryzalin (+Ory, 20 µM, closed squares), or the actin inhibitor Latrunculin B (+LatB, 2 µM, closed triangles) in *V. rupestris* (**E, F**) versus *V. vinifera* cv. ‘Pinot Noir’ (**G, H**) were compared to the solvent control (DMSO, open circles). Representative timelines from five independent series are shown.

The mitogen-activated protein kinase (MAPK) cascades represent one of the major signalling systems of eukaryotic cells. Several MAPK cascades were shown to be associated with the induction of plant defence responses [45],[46]. To understand, why alkalinisation is transient, we probed for a possible feedback of MAPK signalling using PD98059, a specific inhibitor of the MAPK cascades. For flg22-triggered alkalinisation, we observed a conspicuous pH-response which decreased gradually after a peak at 20 min, and the inhibitor significantly reduced the slope of decrease resulting in an almost stable alkalinisation in *V. rupestris* (Figure 2A). For Harpin-triggered alkalinisation that was already constitutive in *V. rupestris*, it was not possible to increase pH even further by treatment with PD98059 (Figure 2B). However, flg22 slightly enhanced the pH-response in *V. vinifera* cv. ‘Pinot Noir’, although the amplitude remained very low (Figure 2C). In contrast, here the transient Harpin-triggered alkalinisation could be rendered constitutive by 100 µM of PD98059 in *V. vinifera* cv. ‘Pinot Noir’ (Figure 2D). This means that, in this case, inhibition of the MAPK cascades in the less responsive *V. vinifera* cv. ‘Pinot Noir’ line almost phenocopied the constitutive pH response in the responsive *V. rupestris*. These findings indicate that the transient nature of elicitor-triggered alkalinisation is caused by a negative feedback from (downstream) MAPK signalling. This negative feedback is more pronounced in Harpin-triggered signalling, and it is more relevant in *V. vinifera* cv. ‘Pinot Noir’.

### The cytoskeleton modulates alkalinisation

In addition to its role in the machinery driving cell division and expansion, the cytoskeleton acts as a sensor for environmental stimuli through a mechanosensitive activity at the plasma membrane [47]. To investigate, whether the organisation of the cytoskeleton modulates the alkalinisation induced by flg22 or Harpin, Oryzalin, an inhibitor of microtubule polymerisation specific for plants, and Latrunculin B, impeding the assembly of actin filaments, were used in this study. In both cell lines, application of Oryzalin significantly (up to ∼0.4 pH units in *V. rupestris*) decreased the amplitude of alkalinisation for both flg22- (Figures 2E, G) and Harpin-elicitation (Figures 2F, H). In a control experiment, the same concentration of Oryzalin caused a small alkalinisation of ∼0.1 in *V. rupestris*, and of ∼0.05 in *V. vinifera* cv. ‘Pinot Noir’ (Figure S1 in supporting information). In contrast, Latrunculin B caused a small, but significant elevation (about ∼0.1 pH units) of alkalinisation in *V. rupestris* for both elicitors (Figures 2E, F). In *V. vinifera* cv. ‘Pinot Noir’, this elevation was not observed (Figures 2G, H). In case of Harpin, Latrunculin B even caused a significant suppression of alkalinisation (Figure 2H). Here, a control with the same concentration of Latrunculin B in the absence of elicitor caused a slight alkalinisation that remained insignificant (Figure S1 in supporting information). These results demonstrate that microtubules act as positive modulators of alkalinisation, whereas actin constrains alkalinisation in the responsive *V. rupestris* line (but not in the less responsive *V. vinifera* cv. ‘Pinot Noir’).

### Oxidative burst is induced differently by flg22 and Harpin

**Figure 3:**
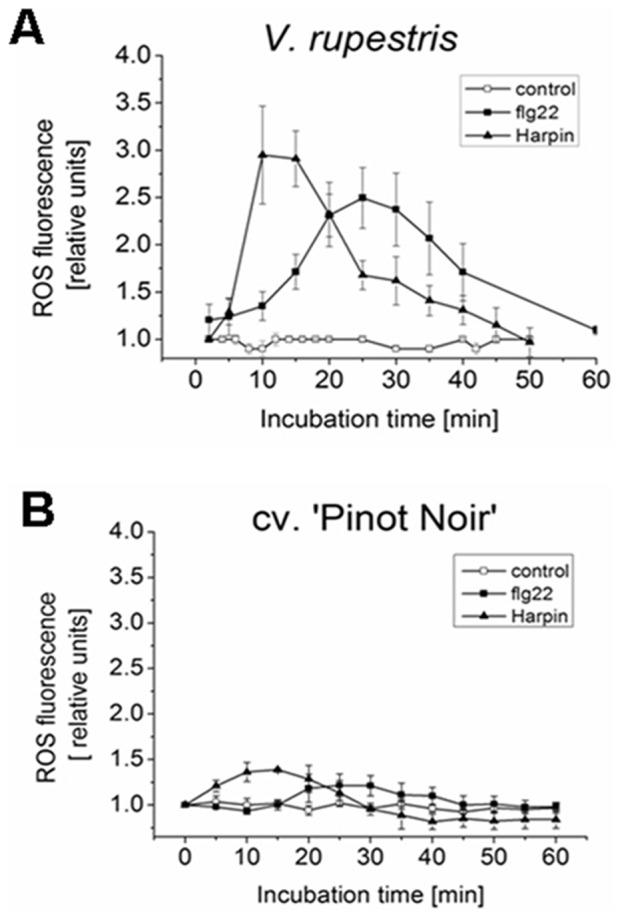
Production of reactive oxygen species (ROS) triggered by flg22 and Harpin. Time-course of ROS accumulation monitored by dihydrorhodamine 123 (DHR 123) in response to the water control (open circles), flg22 (1 µM, closed squares), or Harpin (9 µg^.^ml^−1^, closed triangles) in *V. rupestris* (**A**) versus *V. vinifera* cv. ‘Pinot Noir’ (**B**). Relative fluorescence recorded at constant exposure time (100 ms) was quantified relative to the respective base fluorescence by the Image J software as described in Material and Methods.

The rapid generation of reactive oxygen species (ROS), termed oxidative burst, is induced early during pathogen invasion or elicitor treatment [48]. To test, to what extent oxidative burst is triggered by flg22 or Harpin, we used the fluorescent dye dihydrorhodamine 123 (DHR 123) to follow ROS production after incubation with either flg22 (1 µM) or Harpin (9 µg^.^ml^−1^) as compared to a water control. No significant changes were observed for the solvent control in the two cell lines (Figure 3). However, fluorescence was pronouncedly elevated after both flg22 and Harpin treatments in both cell lines. In *V. rupestris* (Figure 3A), the signal increased immediately to a transient peak of about 3.0 fold at 10–15 min after Harpin elicitation and then dropped back rapidly, whereas flg22-induced ROS production initiated with a delay of about 15 min with a peak of about 2.5 fold signal at 25–30 min and a subsequent decrease (Figure 3A). In contrast to *V. rupestris*, in *V. vinifera* cv. ‘Pinot Noir’ oxidative burst although occurring with similar time courses as for *V. rupestris* was much weaker with only slight inductions of 1.4 fold for Harpin and 1.2 fold for flg22 application, respectively (Figure 3B). In summary, we observed that both flg22 and Harpin induced only a transient oxidative burst, indicating that these ROS act as signal rather than as components of the machinery executing hypersensitive cell death [49]. This early oxidative burst happens significantly earlier in case of Harpin elicitation as compared to flg22.

### Flg22 and Harpin induce expression of defence genes in a similar way

**Figure 4:**
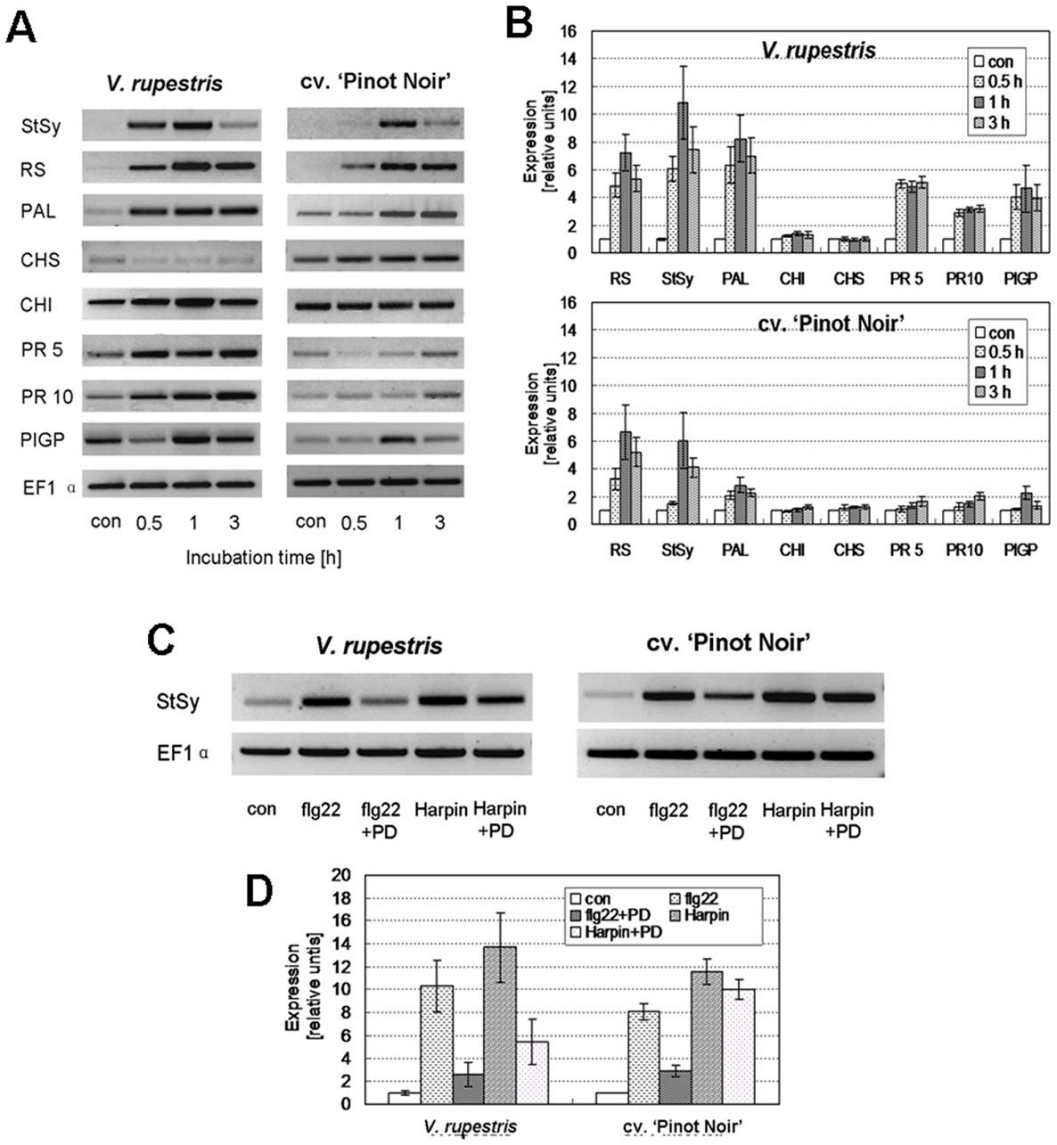
Expression of defence-related genes induced by flg22. **A, B** Representative gels showing transcript abundance followed by semi-quantitative RT-PCR after elicitation with 1 µM flg22 (**A**), and quantification relative to elongation factor 1α (**B**) as reference. The data represent mean values from three independent experimental series; error bars show standard errors. Genes of interest encode proteins including PAL, phenylalanine ammonium lyase; CHS, chalcone synthase; StSy, stilbene synthase; RS, resveratrol synthase; and CHI, chalcone isomerase; pathogenesis-related proteins: PR10 ad PR5, and PGIP: polygalacturonase-inhibiting protein. **C, D** Influence of MAPK signalling on the abundance of *StSy* transcripts. Cells were challenged by 1 µM flg22, by 9 µg^.^ml^−1^ Harpin (both in the solvent DMSO) alone or in combination with the MAPK cascades inhibitor PD98059 (PD). A representative agarose gel is shown in **C**, the quantification relative to elongation factor 1α from four independent experimental series in **D**, error bars represent standard errors.

The synthesis of phytoalexins and other antimicrobial compounds represents a central element of plant defence. We therefore followed the transcript levels of key players in grapevine defence by semi-quantitative RT-PCR using an elongation factor 1α gene (EF 1α) as internal standard. The transcription activation of the flavonoid pathway was monitored by probing for phenylalanine ammonium lyase (PAL), chalcone synthase (CHS), and chalcone isomerase (CHI), the stilbene pathway by stilbene synthase (StSy) and resveratrol synthase (RS), and the activation of pathogenesis-related proteins by probing for PR5, and PR10, and the polygalacturonase-inhibiting protein (PGIP) [50],[51],[52]. Compared to the results obtained using the Harpin elicitor published previously by Qiao *et al*. [33], the gene expression profile induced by flg22 was similar. In control cells, no significant transcript accumulation of genes was detected during the incubation period. Similar to elicitation by Harpin [33], the flg22-response was faster and stronger in *V. rupestris* than in *V. vinifera* cv. ‘Pinot Noir’ (Figures 4A, B). In *V. rupestris*, the transcripts of *StSy* and *RS*, driving stilbene biosynthesis, accumulated from 30 min, peaked at 1 h, and had decreased at 3 h, whereas in *V. vinifera* cv. ‘Pinot Noir’ at 30 min any accumulation was hardly detectable. Similarly, flg22 induced a higher expression of *PAL*, and *PGIP*, whereas there was not significant up-regulation for *CHS* and *CHI*. Expression of *PR*10 and *PR*5, important factors for pathogen-susceptibility and hypersensitive cell death [53], were induced strongly and rapidly in *V. rupestris*, but only weakly, if at all, in *V. vinifera* cv. ‘Pinot Noir’. The transcript patterns observed after treatment with flg22 were very similar to those triggered by Harpin [33]. It was shown that both flg22 and Harpin induced defence gene expression in a similar way. Thus, flg22 and Harpin, although activating different levels of immunity, seem to activate comparable patterns of defence-related genes.

### MAPK activity is necessary for flg22, but not for Harpin-induced *StSy* transcription

The MAPK cascades have also been implied in the activation of defence gene expression in several studies [45]. To test, whether this signalling pathway, in addition to its feedback regulation of alkalinisation (Figures 2A–D), is involved in the activation of defence genes, we investigated the transcription of *StSy* as representative example and used the MAPK cascades inhibitor PD98059. Analysis of semi-quantitative RT-PCR showed that PD98059 in both cell lines partially inhibited *StSy* expression triggered by either flg22 or Harpin (Figures 4C, D). However, the inhibition was much stronger for flg22-induced, much weaker for Harpin-induced *StSy* transcription. A comparison of flg22-induced transcript abundance between the cell lines showed that the inhibition was more pronounced in *V. rupestris* over that observed in *V. vinifera* cv. ‘Pinot Noir’. Thus, MAPK signalling is necessary for flg22- triggered transcription of *StSy*, but not so essential for Harpin-triggered transcription, especially in the disease-susceptible *V. vinifera* cv. ‘Pinot Noir’.

### Stilbenes accumulate differently for flg22- versus Harpin-elicitation

**Figure 5:**
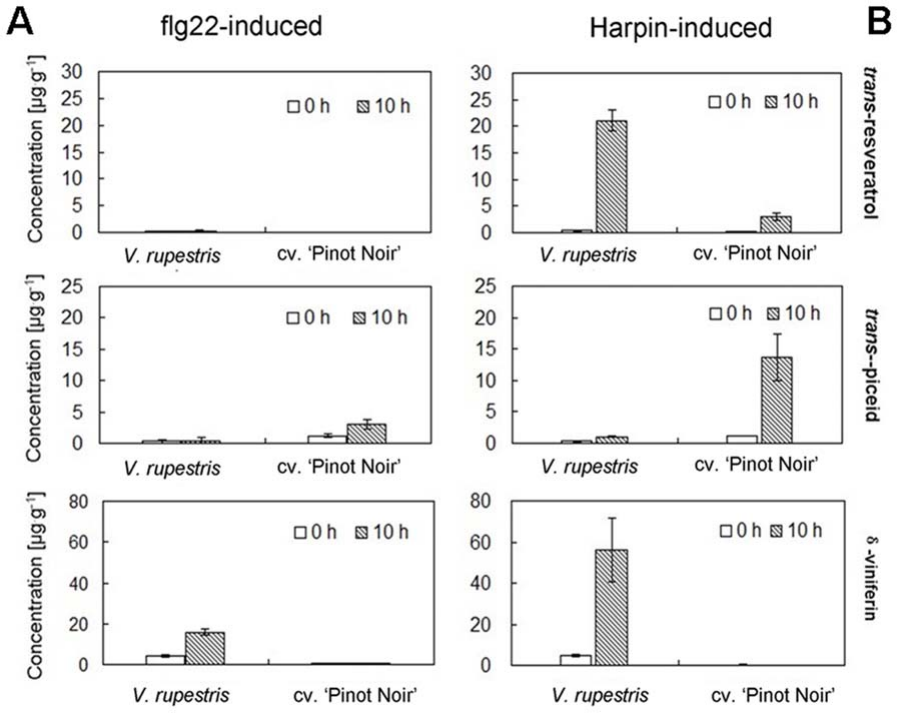
Stilbene accumulation in response to flg22 and Harpin. Cells of *V. rupestris* and *V. vinifera* cv. ‘Pinot Noir’ were exposed to either 1 µM flg22 or 9 µg^.^ml^−1^ Harpin for 0 (white bars) or 10 h (striped bars). Contents of *trans*-resveratrol, *trans*-piceid and δ-viniferin were determined by HPLC and quantified relative to their corresponding calibration curves based on reference standards, respectively. Mean values and standard errors from at least three independent experimental series are shown.

To investigate the effect of flg22 on the enzymatic StSy activity, the products, the stilbenes *trans*-resveratrol, its glucoside *trans*-piceid, and its oxidised dimer δ-viniferin were quantified in both cell lines by HPLC after 10 h incubation with 1 µM flg22 or with 9 µg^.^ml^−1^ Harpin, respectively, as described in our previous study [53]. As shown in Figure 5A, flg22 failed to induce any detectable *trans*-resveratrol in any of the cell lines (Figure 5A, up). The biologically inactive glucoside of resveratrol, *trans*-piceid (Figure 5A, middle), was detectable in low abundance (3.5 µg^.^g^−1^) in *V. vinifera* cv. ‘Pinot Noir’, but was virtually absent in *V. rupestris* (1.17 µg^.^g^−1^). The biologically active oxidative dimer δ-viniferin accumulated to modest 20.76 µg^.^g^−1^ in *V. rupestris*, while there was almost no δ-viniferin detectable in *V. vinifera* cv. ‘Pinot Noir’ (Figure 5A, low). This weak stilbene accumulation in response to flg22 contrasted with the strong accumulation triggered by Harpin (Figure 5B): Here, *V. rupestris* produced high levels of *trans*-resveratrol (21.1 µg^.^g^−1^), and δ-viniferin (56.06 µg^.^g^−1^), but again low levels of *trans*-piceid (1.06 µg^.^g^−1^), In contrast, *V. vinifera* cv. ‘Pinot Noir’ accumulated small amounts of *trans*-resveratrol (2.99 µg^.^g^−1^) and δ-viniferin (0.05 µg^.^g^−1^), but significant amounts of *trans*-piceid (18.5 µg^.^g^−1^). Thus, flg22 and Harpin differ qualitatively in their ability to induce stilbenic compounds, although both can activate *StSy* transcripts to a comparable extent (Figure 4).

### Flg22 can trigger cytoskeletal responses similar to Harpin

Since cytoskeletal reorganisation is associated with the resistance of plant cells to penetration by pathogens [54], and since cytoskeletal drugs can modulate apoplastic alkalinisation (Figures 2E–H) and can induce defence genes in the absence of elicitor [33], we investigated the cytoskeletal organisation after treatment with flg22. The response to Harpin had been analysed previously [33],[53].

**Figure 6:**
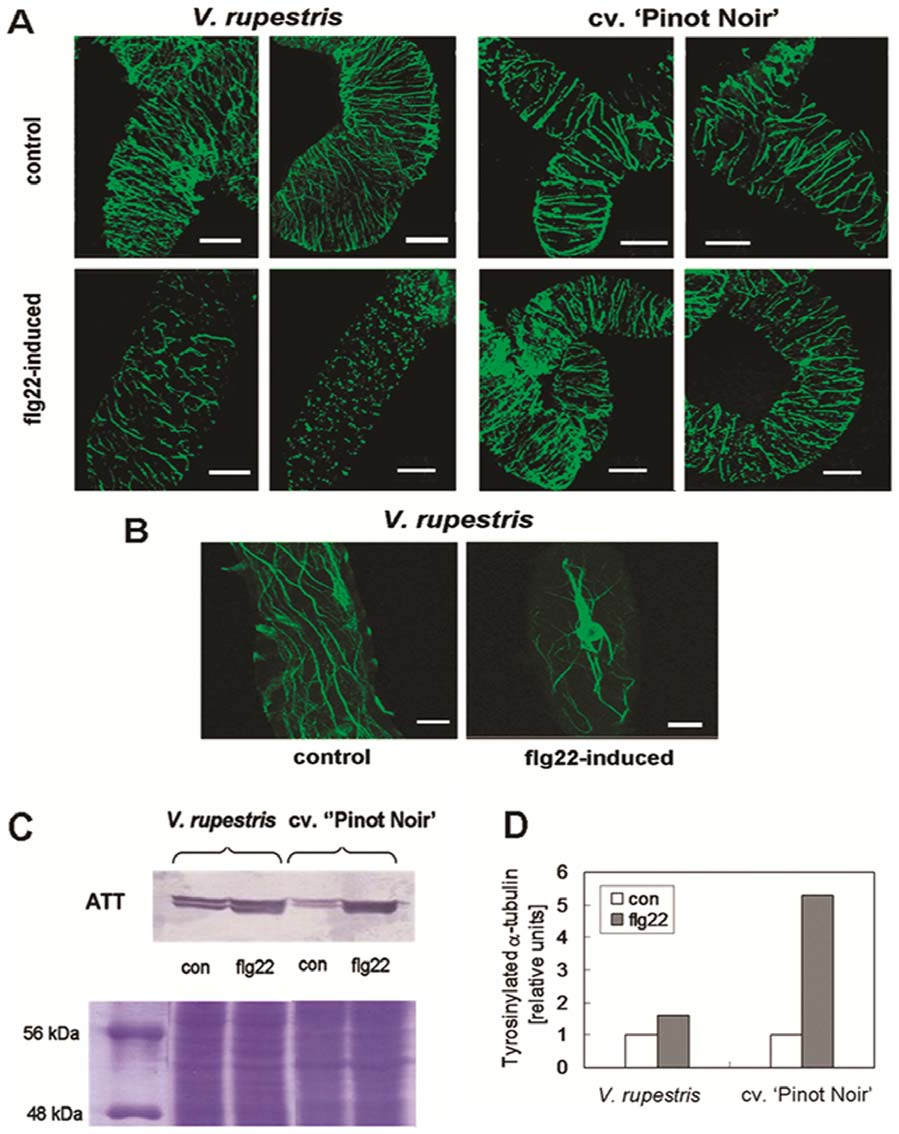
Response of the cytoskeleton to flg22. **A** Disintegration of microtubules visualised by immunofluorescence1 h after addition of 1 µM flg22 or water as negative control. Size bar 20 µm. **B** Reorganisation of actin filaments visualised by FITC-phalloidin upon flg22 treatment as compared to the water control. Representative geometrical projections from Apotome Z-stacks collected from control (left) or after 3 h (flg22-induced, right) of treatment with 1 µM flg22 are shown. Size bar 20 µm. **C** Abundance of tyrosinylated α-tubulin in total extracts 24 h after additioin of 1 µM flg22 visualised by Western blotting probing with specific monoclonal antibodies. The same amount of total protein was loaded in each lane, verified by staining of a replicate by Coomassie Brilliant Blue. **D** Relative abundance of tyrosinylated α-tubulin quantified for the flg22 treatment (flg22, grey bars) as compared to control (con, white bars).

We observed disintegration of microtubules in *V. rupestris* 1 h after treatment with 1 µM flg22, whereas microtubules were only slightly affected in *V. vinifera* cv. ‘Pinot Noir’ (Figure 6A), resembling the situation observed for Harpin [33]. Actin filaments that, in control cells, formed fine strands in the periphery of the cells, became strongly bundled and had contracted towards the nucleus 3 h after incubation with 1 µM flg22 (Figure 6B) again similar to the pattern observed after treatment with Harpin [33],[53].

Since the degree of flg22-induced microtubule disintegration varied between the two *Vitis* cell lines, we wanted to understand whether this difference in the microtubular response was related to a difference in microtulular dynamics reported by the abundance of tyrosinylated α-tubulin probed by the monoclonal antibodies ATT. When soluble proteins from control and flg22-triggered cells were compared, the signal labeled by ATT antibody was strongly increased 24 h after elicitation with flg22 (Figures 6C, D). This response was especially pronounced in *V. vinifera* cv. ‘Pinot Noir’ indicating that here microtubules acquired a higher turnover after treatment with flg22.

### Harpin, but not flg22, can induce cell death

**Figure 7:**
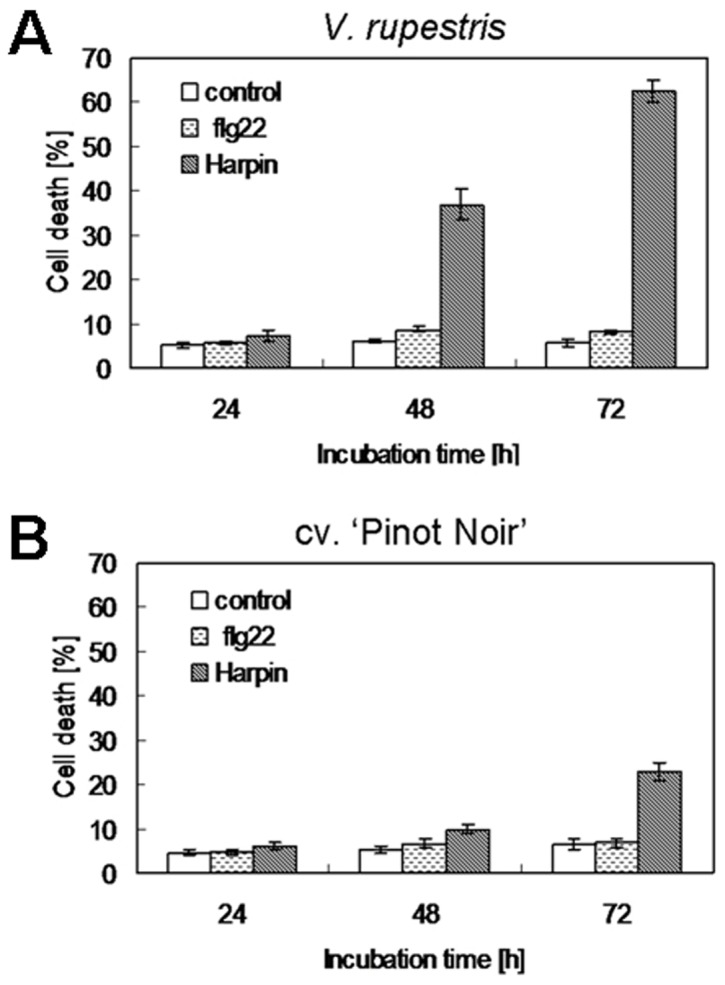
Time course of cell mortality in response to flg22 and Harpin. The relative frequency of dead cells after treatment with flg22 (1 µM, dotted bars) or Harpin (9 µg^.^ml^−1^, shaded bars) as compared to the water control (white bars) in *V. rupestris* (**A**) and *V. vinifera* cv. ‘Pinot Noir’ (**B**) was followed over time scoring samples of 1500 cells for each data point. Mean values and standard errors from four independent experimental series are shown.

Activation of defence responses often results in a hypersensitive response (HR) occurring at infection sites which is characteristic in ETI, but rare in PTI [1],[34]. Therefore, we followed cell viability after challenge by flg22 or Harpin using Evans Blue staining. We observed that Harpin induced cell death in both *V. rupestris* and *V. vinifera* cv. ‘Pinot Noir’. In *V. rupestris*, cell death increased strongly from 48 h, and reached more than 60% at 72 h after elicitation (Figure 7A), whereas in *V. vinifera* cv. ‘Pinot Noir’ mortality was much lower with only some 23% at 72 h (Figure 7B). In contrast to Harpin, 1 µM of flg22 did not induce significant mortality in any of the two lines (Figure 7B), although this concentration activated the full repertory of defence responses.

## Discussion

**Figure 8:**
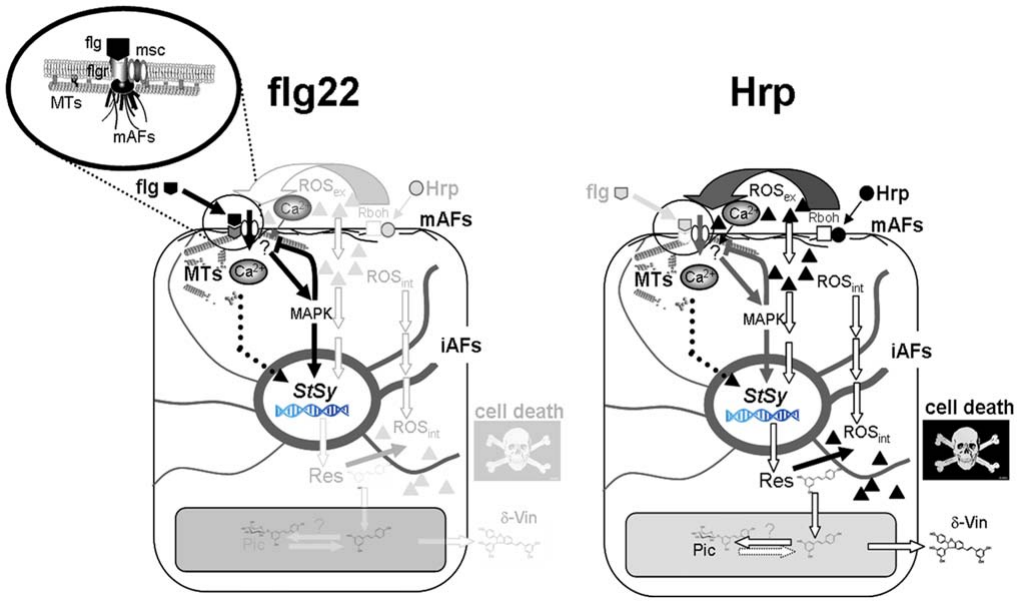
A simplified model for defence triggered by flg22 and Harpin in grapevine cells. Details are explained in the discussion. **Flg**, flg22; **Hrp**, bacterial protein Harpin; **flgr**, flg22 receptor (grapevine homologue of AtFLS2); **msc**, mechanosensitive ion channel; **MTs**, microtubules; **mAFs**, membrane-associated actin filaments; **Rboh**, grapevine homologue of NADPH dependent oxidase responsible for apoplastic oxidative burst (**ROS_ex_**) that can permeate the plasma membrane (**ROS_int_**); **MAPK**, MAPK-signalling pathway; **StSy**, stilbene synthase gene; **iAFs**, intracellular actin filaments; **Res**, *trans*-resveratrol; **δ-Vin**, δ-viniferin; **Pic**, *trans*-piceid.

Plants have developed sophisticated defence systems typically grouped around two levels of immunity, PTI and ETI. A limited set of signal components is organised and integrated to efficiently overcome host or non-host pathogens. However, not all pathogenic defence activators conform to the common signalling mechanism. It therefore is interesting to investigate the immunities triggered by different pathogenic inducers. Accumulating evidence suggests that inducible immunities by PAMPs or effectors often share common signal components. However, at what point these different immunities converge or diverge, is still far from understood. The current models of defence signalling have mainly been driven by hallmark discoveries from the model plant *Arabidopsis thaliana*. It is to be expected that specific aspects from other models will enrich and modify our knowledge of plant immunity. We have therefore employed cell cultures from the disease-resistant grapevine *V. rupestris* and the susceptible grapevine *V. vinifera* cv. ‘Pinot Noir’ to study signal events triggered by the bacterial PAMPs flg22 or Harpin that differ with respect to their ability to trigger cell death. We compared the dependence of apoplastic alkalinisation (as readout for early signalling) on calcium channels, cytoskeleton, and MAPK cascades, and we observed certain differences depending on the nature of the trigger and the cell line. From our observations and previous publications on this system [33],[53], a (simplified) model on defence signalling can be deduced (Figure 8).

### Elicitor perception and apoplastic alkalinisation

Changes in ion fluxes across the plasma membrane, especially increased influx of Ca^2+^ and H^+^, and efflux of K^+^, have been proposed to be part of the signal transduction chain [7]. These can be conveniently measured using apoplastic alkalinisation as readout [55], which allows deriving quantitative data on perception of the respective elicitor. The fact that the apparent affinity of the putative perception system for flg22 is orders of magnitudes higher as compared for Harpin, and the finding that the alkalinisation in response to Harpin is delayed by 5–10 min as compared to flg22 (Figures 1A, B) leads to a model, where the link between flg22 and alkalinisation is more direct, whereas the link between Harpin and alkalinisation is indirect. In our previous work [33], we have shown that the induction of gene expression by Harpin requires apoplastic ROS suggesting that the effect of Harpin on alkalinisation is transduced via an apoplastic oxidative burst, for instance through a grapevine homologue of the NADPH-dependent oxidoreductase Rboh (Figure 8). In *Arabidopsis thaliana*, flg22 is directly recognised by the plasma membrane receptor-like kinase FLS2 that acts together with another receptor-like kinase, BRI-1-associated receptor kinase 1 (BAK1) [15] to activate downstream signalling [7],[10],[14]. A putative grapevine homologue of *AtFLS2* has been identified [56]. So far, there is no direct evidence for a specific host receptor binding Harpin. However, oligomerisation and formation of ionophore-like membrane pores was shown for Hrp7 to depend on a 24-amino-acid motif in the C-terminus, indicating a certain specificity of interaction [57].

### Calcium

Apoplastic alkalinisation is thought to record the activity of a (mechanosensitive) calcium influx-channel [43]. In fact, we observed that alkalinisation was inhibited by GdCl_3_ (Figures 1E–H), but flg22-triggered alkalinisation was much more sensitive as compared to the Harpin-triggered response. This indicates that the flg22-receptor interacts more directly with the calcium influx channels, whereas the ion fluxes triggered by Harpin must involve pathways that do not utilise Gd-sensitive calcium channels. In fact, Harpin has been shown to cause membrane pores that are permeable for cations such as calcium and protons [8]. The signalling target for this calcium influx remains to be elucidated, at least for Harpin it could be shown that it is dispensable for gene activation in tobacco [32].

### Cytoskeleton and early signalling

We tested the role of actin for the apoplastic alkalinisation using the specific inhibitor Latrunculin B. We observed a slight, but significant stimulation of both, flg22- and Harpin-triggered alkalinisation in the responsive *V. rupestris* line (Figures 2E, F) indicating that actin negatively modulates membrane permeability. This finding is consistent with previous findings that actin stabilises plant membranes, probably by releasing membrane tensions through mobilisation of membrane material [58]. In contrast to Latrunculin B, Oryzalin produced a significant reduction of elicitor-triggered alkalinisation (Figures 2E–H) indicating that microtubules are required to activate defence related ion fluxes in response to the elicitors. Oryzalin can activate alkalinisation in the absence of elicitors (followed by a partial activation of defence-related transcription), which can be explained by gating of mechanosensitive calcium channels through microtubules [47]. However, the reduction of flg22- or Harpin-triggered alkalinisation by Oryzalin cannot be explained by removal of the microtubular gating function, but suggests that microtubules somehow help to convey the information of elicitor binding to the channel. Since Oryzalin was added simultaneously with the elicitors and therefore acted only over a short time span, these sensory microtubules must be endowed with high dynamics. A similar transducer function of highly dynamic microtubules has been also observed in other sensory processes such as cold or gravity sensing [47]. Similar to Harpin elicitation, flg22 caused bundling of actin filaments and a fragmentation of microtubules. This microtubular response was hardly detectable in *V. vinifera* cv. ‘Pinot Noir’ but pronounced in *V. rupestris*, and accompanied by an increase of tyrosinylated α-tubulin indicative of a stimulated microtubular turnover (Figure 6). The mechanism for this stimulated microtubular turnover is not known, but it should be mentioned in this context that the MAPK cascade regulates, through its the NACK-PQR branch, the activity of MAP65, an important regulator of microtubular dynamics [59]. An alternative mechanism might involve the microtubule-stabilising protein spiral1 that is recruited for proteasome-mediated degradation in response to osmotic stress [60].

### MAPK signalling

Many stress signals that induce changes in extracellular and/or intracellular pH also activate mitogen-activated protein kinase (MAPK) cascades [61],[62]. Typically, MAPK cascades are composed of three layers: a MAPKKK (MAPK kinase kinase), a MAPKK (MAPK kinase), and a MAPK [46] that can convey signals from upstream kinases to downstream targets including activation of transcription factors, differentiation, cell division, and environmental stresses [63]. In fact, MAPK activity is activated by Harpin in cells of *Arabidopsis thaliana* and tobacco [45],[64], and flg22 treatment triggers a rapid phosphorylation of proteins and a transient activation of the MAPK cascade including MPK3/MPK4/MPK6 [65],[66],[12]. To avoid constitutive overstimulation of defence signalling, the primary signals have to be switched off, once the signal has been transferred to intracellular acceptors. For instance, the flg22 receptor FLS2 is internalised following binding of the ligand [67]. Alternatively, the activity of the triggering ion channel could be downregulated by negative feedback from downstream signals. In fact, we observe that PD98059, an inhibitor of MAPK signalling can render a transient alkalinisation (in *V. vinifera* cv. ‘Pinot Noir’) into a constitutive signal (Figure 2C) suggesting that MAPK signalling produces such a negative feedback avoiding overstimulation of defence. In addition to this feedback, MAPK signalling is required for the activation of *StSy* transcription, a central player of phytoalexin synthesis (Figures 4C, D), but seems to be more essential for the transduction of flg22, whereas the Harpin signal seems to be transduced in parts independently of MAPK signalling. This contrasts with findings in tobacco, where Harpin triggered the *PR*-gene *HIN1* through calcium-independent MAPK signalling [32]. Thus, the exact link between calcium influx, activation of MAPK signalling and gene activation warrants further investigation.

### Activation of defence genes

We tested a panel of defence-related genes that are activated by Harpin in both grapevine cell lines [33] for their response to flg22 elicitation (Figures 4A, B). Although we found differences between the cell lines (a weaker response of *V. vinifera* cv. ‘Pinot Noir’), the pattern was fairly similar to that obtained for Harpin elicitation. In *Arabidopsis thaliana*, it was observed that the PAMP flg22 and the effector Avr9 activated a substantially overlapping set of genes [68].

### Oxidative burst

Oxidative burst has a dual function in defence, either as early stress signal or as part of the downstream machinery that attacks invading pathogens [69]. The rapid and transient production of ROS production in response to elicitors is dependent on a NADPH oxidase [70]. In our grapevine system, we observed a distinct difference in timing of oxidative burst induced by flg22 and Harpin (Figure 3). Whereas Harpin triggered an early oxidative burst (preceding alkalinisation), the oxidative burst triggered by flg22 was later (and follows alkalinisation and even activation of defence-related transcripts). This means that the oxidative burst in flg22 cannot act as an early signal, but rather represents a downstream response. In contrast, Harpin signalling seems to employ oxidative burst. In our previous work, we have shown for the grapevine cell system that apoplastic ROS are necessary for the induction of *StSy* by Harpin [53].

### Stilbene synthesis

The products of stilbene synthase/resveratrol synthase (StSy/RS), the stilbene resveratrol, are a class of phytoalexin produced by plants as part of the defence response. In grapevine, resveratrol efficiently blocks pathogens such as Downy and Powdery Mildew [71],[38]. In addition to resveratrol, its metabolic compounds are endowed with high antimicrobial activity and accumulate in grapevine as a result of infection or stress [37],[72],[38],[73]. Among those metabolic compounds, oxidised δ-viniferin is even more toxic than resveratrol itself and capable of inhibiting zoospore mobility of *Plasmopara viticola*, whereas the glucoside piceid shows no or little toxicity and no antimicrobial activity [74],[75]. Although in the two cell lines both, flg22 and Harpin induced the *StSy* transcript to a similar degree (Figures 4A, B), the educts of stilbene synthesis, resveratrol, and its oxidised dimer δ-viniferin accumulated to significant amounts only in response to Harpin elicitation (Figure 5) in *V. rupestris*, whereas flg22 only induced marginal levels of δ-viniferin. The inactive glucoside *trans*-piceid was formed instead in *V. vinifera* cv. ‘Pinot Noir’, again, only Harpin can induce significant levels, whereas flg22 was almost inactive. The reason for this difference between the two elicitors remains unknown. The substrate of StSy/RS is also used by chalcone synthase (CHS), a key enzyme of flavonoid synthesis. StSy/RS has originated from CHS via gene duplication and mutation [76]. Since CHS is also induced by flg22, it is conceivable that it diverts the substrate from StSy – however, CHS is also induced by Harpin to a similar degree [33]. This indicates that the balance between StSy and CHS activity might be regulated and partitioned on the posttranslational level. Resveratrol acts as important amplifier of oxidative burst [53] in these grapevine cell lines. This means that Harpin is expected to produce a resveratrol-induced second wave of oxidative burst that is absent in *V. vinifera* cv. ‘Pinot Noir’. Although resveratrol and δ-viniferin are considered as the pivotal phytoalexins, it should be kept in mind that additional stilbenes that have not been addressed in the present study could be relevant as well. We therefore have launched a metabolomics approach to identify additional potential players in the stilbenoid pathway.

### Cell death

Defence responses, in many cases, are accompanied by HR-type programmed cell death, especially in ETI. *V. rupestris* originates from North America, and has evolved sympatrically with several of the major grapevine diseases. Its disease resistance has been intensively studied in the context of resistance breeding and linked with a pronounced capacity for hypersensitive cell death [77] correlated with the *Rpv3* locus, probably encoding a receptor for oomycete effectors [78]. In fact, elicitation by Harpin can trigger pronounced cell death in *V. rupestris*, and to a weaker extent, in *V. vinifera* cv. ‘Pinot Noir’, whereas flg22 was completely ineffective with respect to cell death (Figure 7). Preliminary assays using the TdT-mediated dUTP nick end labeling (TUNEL) assay indicate that the Harpin-triggered response classifies for a HR-type PCD event. However, recent studies emphasise that other forms of cell death, such as autophagy, need to be taken into consideration as well [79].

When the cellular responses triggered by flg22 and Harpin in this and our previous studies are compared, apoplastic alkalinisation, cytoskeletal responses, and calcium influx are triggered by both flg22 and Harpin, although differing in amplitude between *V. rupestris* and *V. vinifera* cv. ‘Pinot Noir’. However, there is evidence for a stricter dependency of *StSy* transcriptional activation on MAPK signalling in case of flg22 elicitation, whereas in case of Harpin signalling, MAPK seems to be at least partially dispensable, indicating a parallel signal pathway. The primary steps of both pathways differ of course: flg22 triggered signalling involves binding of the PAMP to a receptor protein (probably the grapevine homologue of AtFLS2) with high affinity. Harpin activates at much lower affinity and probably not through a receptor protein [57]. A second qualitative divergence of the pathways becomes manifest in oxidative burst: Whereas Harpin causes an early wave of ROS (preceding apoplastic alkalinisation) that is later followed by a second wave of ROS triggered by the stilbene resveratrol [53], flg22 triggers only a sluggish oxidative burst (following apoplastic alkalinisation) and fails to induce formation of resveratrol and thus the signal that produces the second wave of ROS. Since the induction of *StSy* by Harpin seems to be at least partially independent of MAPK signalling, a straightforward hypothesis would assume that it is triggered by a parallel ROS-dependent pathway (Figure 8). It has to be tested, whether the same ROS-dependent pathway is also responsible for the formation of resveratrol and thus for the second wave of oxidative burst correlated with the induction of osmotin-type pathogenesis-related protein 5 and cell death observed in Harpin-elicited *V. rupestris* [53].

The comparison between the cellular responses to flg22 and Harpin provides new insight into defence signalling in *Vitis*-pathogen interactions. Harpin induced cell death in *V. rupestris*, and thus mimicks an ETI situation. On the other side, for several reasons Harpin proteins rather qualify as PAMPs. Unlike canonical effectors that have evolved from a specific interaction between host and pathogen, Harpin proteins are widespread among bacteria and contribute to virulence, probably by contributing to the functionality of the pilus. A further argument against a canonical effector function is the lack of any identified host receptor for Harpins. In fact, the conceptual dichotomy between PTI and ETI, has been softened recently, and certain PAMPs seem to be, in fact, able to cause programmed cell death [6],[34].

We can conclude that some of the early defence responses are shared by flg22 and Harpin signalling and only differ in amplitude, not in quality. We could pinpoint essentially five aspects, where flg22- and Harpin-triggered events differed qualitatively: (i) perception by high-affinity binding to a specific receptor protein in case of flg22, but by low-affinity membrane attachment in case of Harpin, (ii) the early oxidative burst observed within 10–15 min after challenge with Harpin, was delayed by about 15 min in response to flg22, (iii) the accumulation of *StSy* transcripts that required functional MAPK signalling in response to flg22, was mostly independent from MAPK signalling in response to Harpin, (iv) although both elicitor activated *StSy* transcription to a similar extent, the enzymatic products resveratrol and its oxidised derivative δ-viniferin accumulated only in response to Harpin, not in response to flg22, (v) cell death was triggered by Harpin, but not by flg22. These findings suggest that the early defence responses triggered by the flg22 and Harpin, on the one hand, share common signal elements, but differ in a chain of events running in parallel with this shared signalling. This specific parallel signalling is integrated differently at a later stage resulting in a qualitatively different output of defence: basal immunity (*bona fide* PTI) versus cell-death related immunity. To what extent the Harpin-triggered cell-death related immunity overlaps with canonical ETI will be the target of further investigations.

## Materials and Methods

### Cell culture and treatments

Suspension cell cultures of *V. rupestris* and *V. vinifera* cv. ‘Pinot Noir’ established from leaves [33] were maintained in liquid MS medium containing 4.3 g^.^l^−1^ Murashige and Skoog salts (Duchefa, Haarlem, The Netherlands), 30 g^.^l^−1^ sucrose, 200 m g^.^l^−1^ KH_2_PO_4_, 100 mg^.^l^−1^ inositol, 1 mg^.^l^−1^ thiamine, and 0.2 mg^.^l^−1^ 2,4-dichlorophenoxy-acetic acid (2,4-D), pH 5.8. Cells were sub-cultured weekly by transferring 10 ml stationary cells into 30 ml fresh medium in 100 ml Erlenmeyer flasks and then incubated at on an orbital shaker (KS250 basic, IKA150 Labortechnik, Staufen, Germany) at 150 rpm, 25°C, in the dark.

The bacterial peptide flg22 was synthesised by GenScript and diluted in sterile H_2_O. A commercially available Harpin elicitor [Messenger, EDEN Bioscience Corporation, Washington, USA, active ingredient: 3% (w/w) Harpin protein] was prepared into 300 mg^.^ml^−1^ stock solution.

### Measurement of extracellular alkalinisation

Extracellular alkalinisation was measured by combining a pH meter (Schott handylab, pH 12) with a pH electrode (Mettler Toledo, LoT 403-M8-S7/120) as described in Qiao *et al.* [33]. Before addition of elicitors, cells were preequilibrated on an orbital shaker for at least 1 h. The maxima of the pH response were plotted against the flg22 concentration and fitted using a Michaelis-Menten equation: f(x) = ΔpH_max_×x/(EC_50_+x), with ΔpH_max_ as *V_max_*, EC_50_ (the half-maximal pH response) as K_m_, and the concentration of flg22 as [S].

To test for the impact of calcium influx on flg22- or Harpin-dependent extracellular alkalinisation, an inhibitor of mechanosensitive calcium channels, GdCl_3_, was used. Cells were co-incubated with 1 µM flg22, 9 µg^.^ml^−1^ Harpin, either with or without 20 µM of GdCl_3_, a concentration derived from our previous work [33]. To assess the effects of cytoskeletal drugs on flg22- or Harpin-dependent extracellular alkalinisation, microtubules were eliminated by 20 µM of Oryzalin, actin filaments by 2 µM of Latrunculin B added together with flg22 or Harpin as compared to the same volume of DMSO as a solvent control. To examine the influence of MAPK signalling, the inhibitor PD98059 targeted to the mitogen-activated protein kinase kinases (MAPKKs) [63] was added to the cells in variable concentration in DMSO with either flg22 or Harpin.

### Quantification of oxidative burst

The production of ROS was determined by dihydrorhodamine 123 (DHR 123) as previously described [53]. Aliquots of 200 µl suspension were (at day 4 after sub-cultivation) diluted into 800 µl of PBS, pre-equilibrated on a shaker for 1 h and then supplemented with dihydrorhodamine 123 (DHR 123 in DMSO, final concentration 10 µM). After incubation for 30 min, cells were washed 3 times using pre-warmed PBS at 37°C and resuspended in 1 ml PBS in combination with either with 1 µM flg22, with 9 µg^.^ml^−1^ Harpin, or with a corresponding concentration of the solvent (water) as negative control. Changes of the fluorescent signal were followed over time under an AxioImager Z.1 microscope (Zeiss, Jena, Germany) equipped with an ApoTome microscope slider for optical sectioning and a cooled digital CCD camera (AxioCam MRm, Zeiss, Jena, Germany) using the filter set 38 HE (excitation at 470 nm, beamsplitter at 495 nm, and emission at 525 nm), a 20× objective and a constant exposure time of 100 ms. Production of ROS fluorescence was quantified as mean pixel intensity of each image at indicated time points in relation to the corresponding image recorded at 0 min using an Image J software (http://rsbweb.nih.gov/ij/). Error bars represent standard errors from three independent experiments.

### Expression analysis

The abundance of defence-related transcripts was measured by semi-quantitative RT-PCR. Total RNA was extracted and cDNA was synthesised after treatment with 1 µM flg22 for 0.5, 1, 3 h, or with water as control as previously described [33],[53]. Transcripts were amplified by RT-PCR using the primers listed in the Table S1 probing for genes encoding phenylalanine ammonium lyase (PAL), chalcone synthase (CHS), stilbene synthase (StSy), resveratrol synthase (RS), chalcone isomerase (CHI), pathogenesis-related proteins (PR10 and PR5), and polygalacturonase-inhibiting protein (PGIP) [50]–[52]. Values for relative transcript abundance were calculated using elongation factor 1α [51] as internal standard. The data from the quantification represent the mean from at least three independent experimental series.

To determine the influence of mitogen-activated protein kinase (MAPK) cascades on the expression of the marker gene *StSy*, cells were treated with either flg22 or Harpin alone or in combination with the MAPK cascades inhibitor PD98059 (100 µM) for 1 h. Experiments were performed in three biological replicates as described above.

### Quantification of stilbene biosynthesis

To test whether the transcript of stilbene synthase (*StSy*) was accompanied by the final product generated by this enzyme, cells were challenged either with 1 µM flg22, or 9 µg^.^ml^−1^ Harpin for 10 h, a time point chosen according to time-course studies on stilbene synthesis in the same system [53] as compared to a water control. Cells were drained from culture medium by a vacuum of 800 pa (Vacuubrand CVC2, Brand, Germany), frozen in liquid nitrogen, and then stored at −80°C until further analysis. Aliquots of 3 g fresh weight of untreated control or treated cells were homogenised with 20 ml of 80% (v/v) methanol in water by an ultrasonic processor (UP100H, Hielscher, Germany) for 3 min. The homogenate was incubated for 2 h in the dark at room temperature in a rotatory shaker and filtered through filter paper by vacuum with 500 pa. The filtrate was concentrated to a residual volume of 5 ml in a glass tube at 40°C (Heating Bath B490, BÜCHI, Germany) at 280 rpm (Rotavapor R-205, BÜCHI, Germany), under a vacuum of 80 Pa (Vacuubrand CVC2, Brand, Germany). Stilbenes were extracted from the aqueous phase by adding 2 ml of 5% (w/v) NaHCO_3_, and three aliquots of 5 ml ethyl acetate. The pooled ethyl acetate phase was completely dried and the residue suspended in 2 ml of methanol prior to injection into the HPLC.

Analysis of stilbenes was carried out on a high performance liquid chromatograph, HPLC (Agilent, 1200 series, Waldbronn, Germany) as described previously [53]. *Trans*-resveratrol, *trans*-piceid, and δ-viniferin were quantified using external standards on the basis of retention time and UV-VIS spectra. The standards for *trans*-resveratrol (Sigma-Aldrich, Deisenhofen, Germany), *trans*-piceid (Phytolab, Vestenbergsgreuth, Germany) and δ-viniferin (kind gift of Dr. Kassemeyer, State Institute of Viticulture, Freiburg) were dissolved in methanol at a concentration of 100 mg^.^l^−1^. Calibration curves for quantification of the samples were determined using these standards and found to be linear (r^2^>0.99). At least three independent experimental series were conducted.

### Visualisation of cytoskeleton

The response of the cytoskeleton to 1 µM flg22 was assessed using fully expanded cells (day 10 after subcultivation) as compared to a negative control with the corresponding concentration of solvent (water). Microtubules were stained by indirect immunofluorescene using a monoclonal antibody against α-tubulin (DM1A; Sigma-Aldrich, Deisenhofen; Germany), and a secondary anti-mouse IgG antibody conjugated to fluorescein isothiocyanate (FITC; Sigma-Aldrich, Deisenhofen; Germany) following the protocol published previously [33]. Cells were visualised using an AxioImager Z.1 microscope (Zeiss) equipped with an ApoTome microscope slider through the filter sets 38 HE (excitation at 470 nm, beamspliter at 495 nm, and emission at 525 nm).

Actin filaments were visualised with FITC-phalloidin as described previously [80]. Cells were fixed in 1.85% (w/v) paraformaldhyde in 0.1 M PIPES, pH 7.0, supplemented with 5 mM MgCl_2_ and 10 mM EGTA) for 30 min at 25°C. Subsequently, samples were stained with 0.66 µM FITC-phalloidin (Sigma-Aldrich, Deisenhofen, Germany) for 30 min. Cells were then washed three times for 5 min in PBS and observed immediately by an ApoTome microscopy as described above.

### Quantification of tyrosinated α-tubulin

10 ml of cells were treated at day 5 after subcultivation with 1 µM flg22 for 24 h and collected by centrifugation for 10 min, 3 000 rpm (Hettich Centrifuge Typ 1300, Tuttlingen, Germany). Soluble proteins were extracted according to Qiao *et al.* [33]. Samples were dissolved in 20 µl of single-strength sample buffer, vortexed, denatured for 15 min at 95°C, and analysed by SDS-PAGE to equalise the loading volume of samples.

Tyrosinated α-tubulin was detected by Western blotting using the monoclonal antibody ATT (Sigma-Aldrich, Deisenhofen, Germany). Signals were visualised by a goat secondary anti-mouse IgA, conjugated with alkaline phosphatase (Sigma-Aldrich, Deisenhofen, Germany) at a dilution of 1∶2500 in TBST with 3% low fat milk powder. Alkaline phosphatase activity was detected by 66 µl of NBT solution (75 mg^.^ml^−1^ Nitroblue-tetrazolium in 75% dimethylformamid) and 33 µl of BCIP solution (50 mg^.^ml^−1^ 5-Bromo-4-chloro-3- indoxylphosphate-p-Tuloidin in 100% dimethylformamid) in 5 ml staining buffer (100 mM Tris-HCl, 100 mM NaCl, pH 9.7) with 1∶10 (v/v) of 500 mM MgCl_2_. A parallel set of lanes loaded in exactly the same manner was stained with Coomassie Brilliant Blue 250 (Sigma-Aldrich, Deisenhofen, Germany) as a loading control. Abundance of tyrosinated α-tubulin after treatment with flg22 was quantified relative to control with the mean value of each lane using Image J software.

### Determination of cell viability

After cells of *V. rupestris* and *V. vinifera* cv. ‘Pinot Noir’ cells had been incubated with flg22 or Harpin for 24, 48 or 72 h, respectively, aliquots (0.2 ml) of each sample were transferred into custom-made staining chambers to remove the medium, incubated in 2.5% (w/v) Evans Blue [81] for 3 min, and then washed with water several times. The frequency of dead cells was scored using a Fuchs-Rosenthal hematocytometer under bright-field illumination. The mortality values were determined from three independent experiments with at least 1500 cells scored for each data point.

## Supporting Information

Figure S1 **Effect of cytoskeletal drugs on extracellular alkalinisation.**
(DOC)

Table S1 **List of oligonucleotide primers used for expression analysis by RT-PCR.**
(DOC)
