# Supplementary material for: Defence Signalling Triggered by Flg22 and Harpin Is Integrated into a Different Stilbene Output in Vitis Cells
Source: PLoS One. 2012 Jul 6;7(7):e40446. doi: 10.1371/journal.pone.0040446 (PMC3391249; doi:10.1371/journal.pone.0040446)
Supplement: Figure S1 — Effect of cytoskeletal drugs on extracellular alkalinisation. (DOC) [file pone.0040446.s001.doc]

**Supporting information**

**Figure S1 Effect of cytoskeletal drugs on extracellular alkalinisation .**

Effect of the microtubule inhibitor Oryzalin (+Ory, 20 μM, closed circles), or the actin inhibitor Latrunculin B (+LatB, 2 μM, closed circles) in *V. rupestris* (**A, C**) and *V. vinifera* cv. ‘Pinot Noir’ (**B, D**) as compared to the DMSO solvent control (open circles). Representative timelines are shown, and the result was reproduced in five independent series.

**
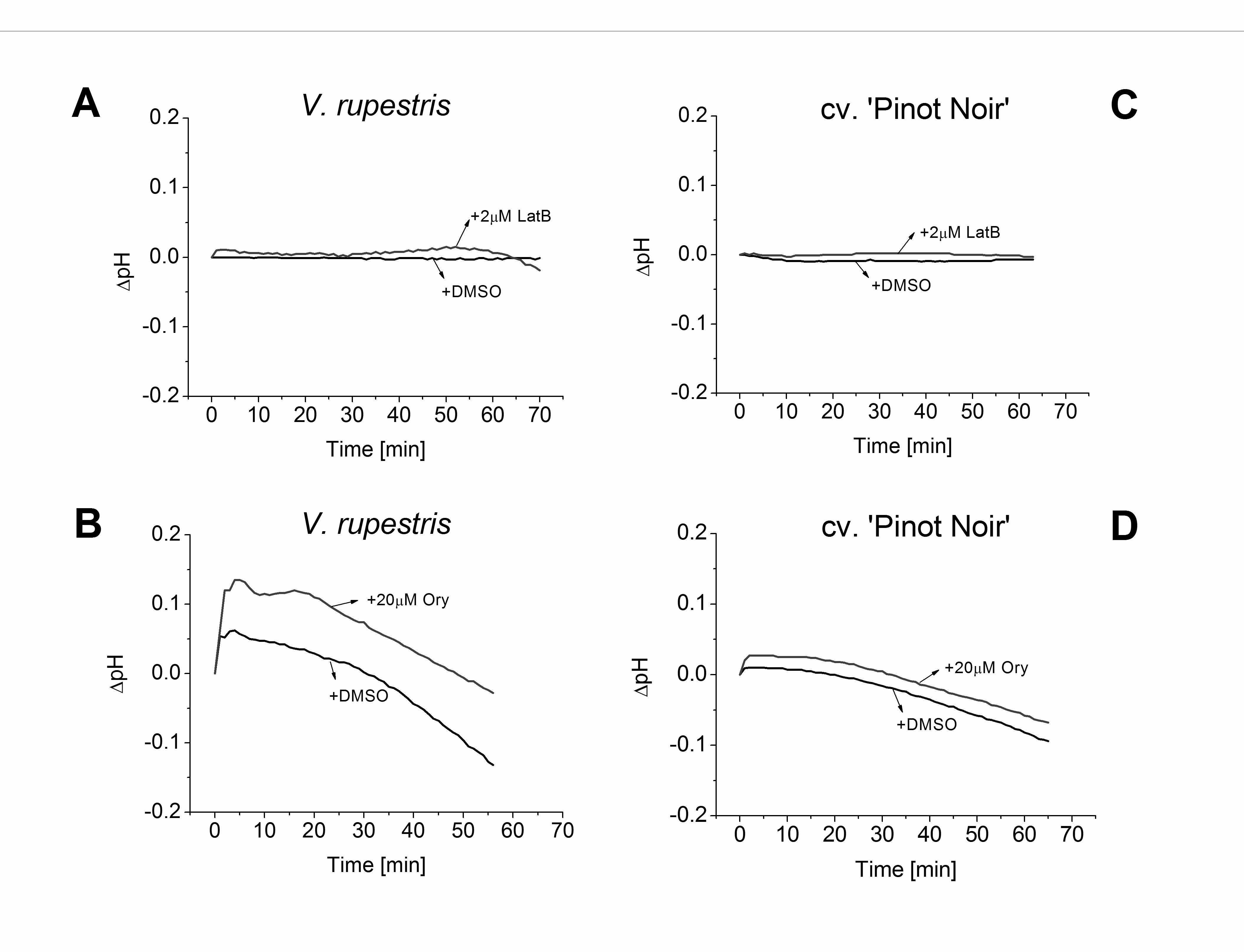
**
