# Supplementary material for: Defence Signalling Triggered by Flg22 and Harpin Is Integrated into a Different Stilbene Output in Vitis Cells
Source: PLoS One. 2012 Jul 6;7(7):e40446. doi: 10.1371/journal.pone.0040446 (PMC3391249; doi:10.1371/journal.pone.0040446)
Supplement: Table S1 — List of oligonucleotide primers used for expression analysis by RT-PCR. (DOC) [file pone.0040446.s002.doc]

**Supporting information**

**Table S1 List of oligonucleotide primers used for expression analysis by RT-PCR.**

| **Name** | **GenBank accession no.** | **Primer sequence 5'-3'** | **Reference** |
| --- | --- | --- | --- |
| EF1α | EC959059 | Sense:5'-GAACTGGGTGCTTGATAGGC-3' Antisense: 5’-AACCAAAATATCCGGAGTAAAAGA-3’ | Reid *et al*. (2006) |
| RS | AF274281 | Sense:5'-GGATCAATGGCTTCAGTCGAG-3' Antisense:5' GCTCCTCAAGCATTTCTTCG 3' | Kortekamp A.(2006) |
| StSy | X76892 | Sense:5'-GAAACGCTCAACGTGCCAAGG-3’ Antisense: 5'-GTAACCATAGGAATGCTATGTAGC-3' | Kortekamp A.(2006) |
| PAL | X75967 | Sense:5’-TGCTGACTGGTGAAAAGGTG-3’ Antisense: 5’-CGTTCCAAGCACTGAGACAA-3’ | Belhadj *et al*. (2008) |
| CHI | X75963 | Sense: 5’-GTTCAGGTCGAGAACGTCC-3’  Antisense: 5’-GCTTGCCGATGATGGACTC-3’ | Kortekamp A.(2006) |
| CHS | AB066274 | Sense:5'-GGTGCTCCACAGTGTGTCTACT-3' Antisense: 5'-TACCAACAAGAGAAGGGGAAAA-3' | Belhadj et al. (2008) |
| PR5 | Y10992 | Sense:5'-CAGCTATGCAGCCACCTTC-3' Antisense: 5'-TCGAAGTTGCAGTTGGTACG-3' | Kortekamp A.(2006) |
| PR10 | AJ291705 | Sense: 5'-CTTACGAGAGTGAGGTCACTTC-3' Antisense: 5'-GCAATAGAACATCACAAATACTCC-3' | Kortekamp A.(2006) |
| PGIP | AF05093 | Sense: 5'-GATGGTACTGCGTCGAATG-3'  Antisense: 5'-GTGGAGCACCACACAAGC-3' | Kortekamp A.(2006) |

EF1α, elongation factor 1α; RS, resveratrol synthase; StSy, stilbene synthase; PAL, phenylalanine ammonia lyase 1; CHS, chalcone synthase; CHI, chalcone isomerase; PR5, PR10, pathogenesis-related proteins 5 and 10, respectively; PGIP, polygalacturonase-inhibiting protein.
